# Genome assemblies of Nuttall’s White-crowned sparrow (*Zonotrichia leucophrys nuttalli)* and Rufous-collared sparrow (*Zonotrichia capensis*)

**DOI:** 10.1038/s41597-025-04889-x

**Published:** 2025-04-03

**Authors:** Zhou Wu, Katarzyna Miedzinska, Jesse S. Krause, Paulina L. Gonzalez-Gomez, Jonathan H. Pérez, John C. Wingfield, Simone L. Meddle, Jacqueline Smith

**Affiliations:** 1 The Roslin Institute and Royal (Dick) School of Veterinary Studies R(D)SVS, The University of Edinburgh, Easter Bush, Midlothian, EH25 9RG UK; 2 Department of Biology, University of Nevada Reno, Reno, NV 89557 USA; 3 Department of Wildlife, Fish and Conservation Biology, University of California Davis, Davis, CA USA; 4 Universidad Autónoma de Chile, Av. Pedro de Valdivia 425, Providencia, Santiago, Chile; 5 Department of Biology, University of South Alabama, Mobile, AL 36688 USA; 6 Department of Neurobiology, Physiology, and Behavior, University of California, Davis, CA 95616 USA

**Keywords:** Genome, Ecological genetics

## Abstract

The *Zonotrichia* sparrows, belonging to the Passerellidae family, are widely studied for their vocalizations and genetic diversity. Here, we present two high-quality genome assemblies for *Zonotrichia* species; the northern hemisphere Nuttall’s white-crowned sparrow (*Zonotrichia leucophrys nuttalli*, NWCS) and the southern hemisphere rufous-collared sparrow (*Zonotrichia capensis chilensis*, RUFS). These assemblies were assembled using PacBio long-reads and Omni-C chromatin conformation technology, integrated with RNA-sequencing data to provide genome annotations. The NWCS assembly comprises 1.12 Gb anchored to 30 chromosomes, while the RUFS assembly includes 1.11 Gb on 27 chromosomes. Both assemblies exhibit high completeness and contiguity, with 96.6% and 96.9% complete BUSCOs (Benchmarking Universal Single-Copy Orthologs) for NWCS and RUFS, respectively. These genome assemblies provide valuable resources for future research on genetic divergence, vocal learning, evolution and responses to climate change within the *Zonotrichia* genus.

## Background & Summary

The *Zonotrichia* sparrows are passerine birds belonging to the family Passerellidae, which are widely distributed across North and South America. The genus *Zonotrichia* consists of 5 sparrow species including *Z. leucophrys*, *Z. albicollis*, *Z. atricapilla*, *Z. capensis*, and *Z. querula*, amongst which only the white-crowned sparrow (*Z. leucophrys*) and white-throated sparrow (*Z. albicollis*) have published genome assemblies. According to the Bird Chromosome Database (V5.0/2024)^1^, the karyotypes of sparrows in the *Zonotrichia* genus are conserved, showing a diploid number of 2n = 82. Recently, we published a chromosome-level reference genome for the Gambel’s white-crowned sparrow (*Zonotrichia leucophrys gambelii*, GWCS)^2^, one of the five sub-species, which offers a foundation to expand our understanding regarding this diverse and widely distributed genus. Due to their widespread distribution and extraordinary vocalizations, *Zonotrichia* (sub-)species are a compelling subject of research study including areas such as hybridization, song learning, animal behaviour, hormones, genetics and evolution^2–7^. They have also been widely studied in relation to environmental change^8–10^. Here, we present genome assemblies for two species within this genus, the non-migratory Nuttall’s white-crowned sparrow sub-species (*Z. leucophrys nuttalli*, NWCS) and the rufous-collared sparrow (*Z. capensis chilensis*, RUFS) of the Southern Hemisphere.

In general, the white-crowned sparrows are mostly distributed across North America, ranging from the Arctic tundra to Rocky Mountains and Pacific coast across northern North America to far eastern Canada. Their wintering range spans from southern parts of the United States into Northern Mexico. White-crowned sparrows are easily recognized by their distinctive head pattern with black and white stripes on the crown, along with a yellow to orange beak. Among the sub-species of white-crowned sparrows, there is diverse variation in seasonal habitat and migratory behaviour. For instance, the NWCS sub-species, that we focus on in this study, is recognized as a permanent coastal resident in coastal California, USA. In contrast, GWCS exhibits long-distance migratory pattern. The sub-species of the white-crowned sparrow exhibit wide divergence across multiple aspects, including sexual behaviours, song variations, demographic histories, environmental adaptations, life-history physiology, as well as genetic differences^3,5,7,11–13^. Importantly, distinct migratory strategy represents one of the most interesting divergences. This may have served as a key driver of behavioural and physiological differences between the sub-species, limiting gene flow and contributing to speciation. Therefore, specific genomic resources offer valuable opportunities to investigate the distinct migratory behaviours observed in *Z. leucophrys*.

The rufous-collared sparrow is primarily distributed from south-eastern Mexico to the southernmost tip of South America, occupying diverse habitats including urbanized areas, deserts, chaparral, forest edge, and alpine meadows^8,14^. There are 27 sub-species of *Z. capensis*, which exhibit different migratory patterns, including long and altitudinal migrants as well as resident sub-species^15,16^. Rufous-collared sparrows are slightly smaller than white-crowned sparrows and exhibit an infamous rufous band around the nape and sides of the neck which contrasts with a grey head with black stripes.

Together with the recently published reference genome assembly of the GWCS, the *Zonotrichia* genome assemblies presented here provide a powerful framework for studying migratory patterns, capturing both within-species and between-species variations. In particular, the subspecies of the white-crowned sparrows exhibit distinct migratory behaviours. While the *Z. capensis chilensis* (RUFS) genome presented here represents an ancestral species that is also a year-round resident and has not been previously sequenced. These phenological differences make the genome assemblies valuable resources for exploring the genomic makeup and genetic drivers underlying migratory patterns. In addition, the contribution of these genome assemblies enables a deeper understanding of comparative genomics (e.g., pangenome graph construction) through utilizing high-quality genetic variants (e.g., structural variants) that are specific to populations. Therefore, these assemblies allow for further exploring the genetic landscapes that underlie divergence and speciation.

To this end, we generated two chromosome-level genome assemblies for the Nuttall’s white-crowned sparrow (*Z. leucophrys nuttalli*) and the rufous-collared sparrow (*Z. capensis chilensis*). We integrated PacBio long-read sequences and chromatin conformation (Omni-C) information to create the genome assemblies, and RNA-sequencing data was used to predict gene models for each assembly. Both genome assemblies exhibit high completeness, yielding 1.12 Gb and 1.11 Gb of sequences anchored to 30 and 27 complete chromosomes for NWCS and RUFS, with 21,727 and 17,845 annotated genes, respectively. These two high-quality genome assemblies of *Zonotrichia* sparrows can advance genomic investigation of this genus. Moreover, the genomic resources of the NWCS sub-species can expand our understanding of genetic divergence and dynamics within specific lineages, as well as potential genetic factors underlying complex phenotypes. The two assemblies provide valuable resources and great opportunities for future genomic research in *Zonotrichia* sparrows.

## Methods

### Sample collection

The sparrows used for genome assemblies were collected from wild free-living populations using mist nets under benign weather conditions. The Nuttall’s white-crowned sparrow (*Z. leucophrys nuttalli*, NWCS) adult female was captured at Point Reyes field site in California, USA, then sedated with isoflurane and euthanized within 3 min. A sample was taken from the pectoralis muscle and immediately frozen and transported on dry ice to the University of California Davis (California, USA). The rufous-collared sparrow (*Z. capensis chilensis*, RUFS) adult female was captured in the vicinity of Fray Jorge National Park, Chile. A 70 µL blood sample was taken within 3 minutes of capture and the bird was released after sampling. The blood sample was centrifuged at 2,000 × g for 5 minutes to separate out the red blood cells. The red blood cells were immediately frozen at −20 °C and shipped on dry ice to the University of California Davis (California, USA). Both muscle and red blood cells were stored at −80 °C until they were shipped on dry ice to Dovetail Genomics (California, USA).

### Genome assembly and sequencing

Chromosome-level genome assemblies were generated for NWCS and RUFS. High molecular weight DNA (50 to 100 Kb) was extracted from the samples for PacBio library preparation. PacBio SMRTbell libraries were constructed using the SMRTbell Express Template Prep Kit 2.0 (PacBio, Menlo Park, CA, USA). Following this, sequencing was performed with PacBio Sequel II 8 M SMRT cells, generating 275 Gb and 170 Gb data for NWCS and RUFS, respectively. Sequences were subsequently assembled into scaffolds using Wtdbg2^17^ with the ‘--genome_size 1.0’ option. Potential contamination in the sequences was identified with Blobtools (v1.1.1)^18^ and haplotypic duplication was purged to resolve haplotig with purge_dups (v1.1.2)^19^. To better resolve the scaffold orientation, a proximity ligation library was generated using the Omni-C technique^15^, followed by sequencing on an Illumina HiSeqX platform (2 × 150 bp, coverage around 30X). The Omni-C library, an alternative to Hi-C and providing superior genomic coverage, uses the sequence-independent endonuclease (DNAse I) for chromatin digestion prior to proximity ligation and library generation. In brief, chromatin was initially fixed in place with formaldehyde, followed by endonuclease digestion. The fragmented chromatin ends were then repaired, biotinylated to adapters, and subsequently subjected to proximity ligation. Finally, the crosslinks were reversed, the biotin was removed and the DNA was purified. Scaffolding of the assemblies was performed using the HiRise pipeline^20^. The pipeline produces a likelihood model to identify the genomic distance and the joins, as well as to resolve the putative mis-joins within the scaffolds. The library preparation, genome sequencing and scaffolding were performed by Dovetail Genomics (California, USA) based on their standard genome assembly pipeline and Omni-C pipeline (https://dovetailgenomics.com/wp-content/uploads/2021/09/Omni-C-Tech-Note.pdf). For genome polishing, additional whole-genome short-read sequence data (round 50X) of one individual from each species was used to make accurate corrections on sequences using POLCA^21^ with default parameters.

### Genome quality assessment and chromosome assignment

**Fig. 1:**
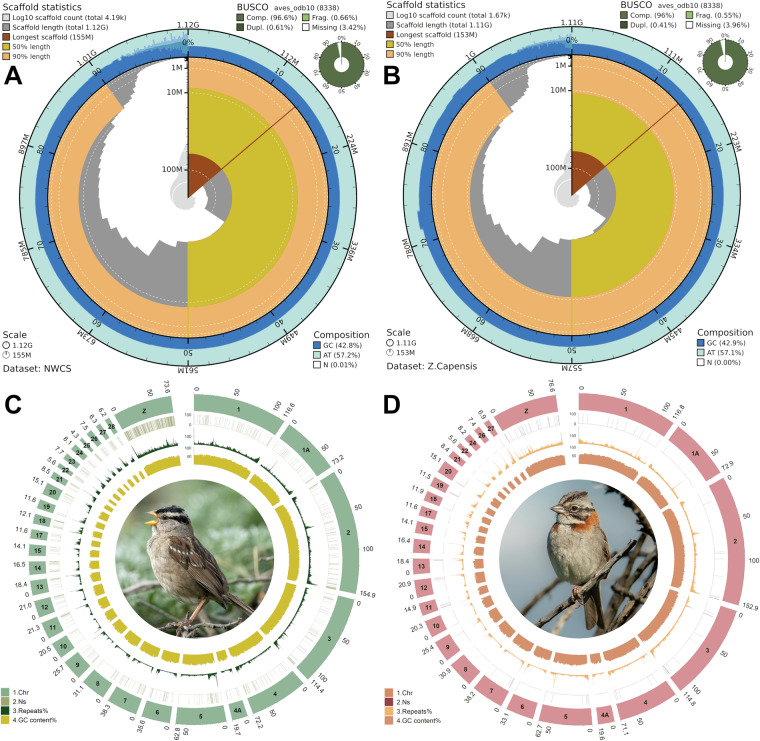
Assessment of the genome assemblies for the white-crowned sparrow (*Zonotrichia leucophrys nuttalli)* and the rufous-collared sparrow (*Zonotrichia capensis chilensis*). Left panels illustrate the genome assembly and scaffold statistics of the Nuttall’s white-crowned sparrow (NWCS) and right panels present for the rufous-collared sparrow (RUFS). (**A,B**) show the snail plots for the two assemblies including a BUSCO (aves_odb10) assessment showing at the top right. (**C,D**) display circos plots of the assemblies, with the following information from outer to inner circles: chromosome length (Mb), Ns, repeat percentage and GC content (window size 200k). The bird photographs were downloaded from Cornell lab of ornithology (https://www.macaulaylibrary.org/, NWCS - ML454295501, RUFS - ML582808131, accessed 2024-Oct-15) under Non-Commercial License Agreement.

The Nuttall’s white-crowned sparrow (*Z. leucophrys nuttalli*) genome assembly yielded a sequence length of 1,121,558,528 bp, comprising 4,194 scaffolds and 5,263 contigs, whereas the rufous-collared sparrow (*Z. capensis chilensis*) genome assembly is 1,113,638,239 bp, comprising 1,673 scaffolds and 1,835 contigs. The overall Ns or gaps in the genome represent 0.009% (107,461 bp) in NWCS and 0.001% (16,951 bp) in the RUFS genome. In addition, the scaffold N50 values for the two assemblies are 72.19 Mb (NWCS) and 71.11 Mb (RUFS) (Table 1). The GC percentage of sequences is 42.77% and 42.89%, for NWCS and RUFS, respectively. The main sequence statistics is demonstrated in snail plots using blobtoolkit (v4.3.11)^18^, where the Benchmarking Universal Single-Copy Orthologs (BUSCO) (v5.2.2)^22^ results are included to provide additional information on the assemblies (Fig. 1A,B). The BUSCO results were analysed in the ‘genome’ mode using the ‘aves_odb10’ lineage dataset. In short, the NWCS and RUFS assemblies exhibit overall complete BUSCOs (C) of 96.6% and 96.9%, respectively, indicating a high level of completeness and contiguity in the assemblies. Furthermore, repeat elements and sequences in the assemblies were estimated and soft-masked in place using RepeatModeler (v2.0.2) and RepeatMasker(v4.1.2)^23,24^. The RepeatModeler was first used to build the genome models and to identify repetitive elements with the ‘-LTRStruct’ parameter to discover long terminal repeat retroelements. Subsequently, RepeatMasker was used to annotate the genome with the ‘-s’ option for a slow but more sensitive search, and ‘-xsmall’ was used to mask repetitive elements in the genome using lowercase letters. In total, 14.63% of the NWCS genome sequences were masked for repeat elements, and 15.15% for the RUFS genome. A detailed profile of the repeat elements in the two assemblies can be found in Table 2. The assemblies were visualized for the chromosome length, N content, GC content, and repeat elements in circos plots using the circlize package (v0.4.15)^25^ in R (v4.1.3)^26^.

**Table 1:** Assessment of two *Zonotrichia* sparrow genome assemblies.

Sequences summary	*Z. leucophrys nuttalli*	*Z. capensis chilensis*
Length of sequences	1,121,558,528	1,113,638,239
Counts of scaffold sequences	4,194	1,673
Largest scaffold length	154,862,712	152,869,446
Scaffold N50	72,190,781	71,112,957
Scaffold L50	6	6
Scaffold N90	7,475,821	11,520,108
Scaffold L90	26	24
GC content (%)	42.77%	42.89%
N Length (bp)	107,461	16,951
N content (%)	0.009%	0.001%
Counts of contigs	5,263	1,835
Maximum length of contigs	23,000,073	69,093,736
Contig N50	3,455,537	16,408,392
Contig L50	94	18
Contig N90	221,062	1,844,731
Contig L90	596	82

**Table 2:** Profile of repeat elements in the Nuttall’s white-crowned sparrow and the rufous-collared sparrow genome assemblies.

Repeats	*Z. leucophrys nuttalli*			*Z. capensis chilensis*		
	Number	Length(bp)	Percentage(%)	Number	Length(bp)	Percentage(%)
Retroelements(total)	245,928	93,378,279	8.33	220,152	86,801,601	7.79
SINEs	2,964	300,979	0.03	2,463	315,007	0.03
Penelope	0	0	0	0	0	0
LINEs	147,706	39,189,962	3.49	133,233	39,267,421	3.53
CRE/SLACS	0	0	0	0	0	0
L2/CR1/Rex	147,280	38,817,847	3.46	132,974	38,893,845	3.49
R1/LOA/Jockey	0	0	0	0	0	0
R2/R4/NeSL	0	0	0	0	0	0
RTE/Bov-B	0	0	0	0	0	0
L1/CIN4	0	0	0	0	0	0
LTR elements	95,258	53,887,338	4.8	84,456	47,219,173	4.24
BEL/Pao	0	0	0	0	0	0
Ty1/Copia	0	0	0	0	0	0
Gypsy/DIRS1	1,174	447,592	0.04	676	230,618	0.02
Retroviral	93,963	53,413,385	4.76	83,693	46,946,047	4.22
DNA transposons(total)	8,785	1,509,135	0.13	6,165	836,260	0.08
hobo-Activator	93	9,609	0	124	23,031	0
Tc1-IS630-Pogo	0	0	0	0	0	0
En-Spm	0	0	0	0	0	0
MuDR-IS905	0	0	0	0	0	0
PiggyBac	0	0	0	0	0	0
Tourist/Harbinger	0	0	0	1,548	200,099	0.02
Other(Mirage,P-element, Transib)	0	0	0	0	0	0
Rolling-circles	1,662	603,340	0.05	2,338	1,782,177	0.16
Unclassified	87,899	48,243,365	4.3	86,881	59,865,826	5.38
Total interspersed repeats		143,130,779	12.76		147,503,687	13.25
Small RNA	517	67,582	0.01	309	90,573	0.01
Satellites	4,878	3,478,308	0.31	5,820	3,084,141	0.28
Simple repeats	236,395	13,720,001	1.22	235,842	13,296,271	1.19
Low complexity	49,496	3,168,177	0.28	48,506	3,007,017	0.27

Finally, the chromosomes were assigned to scaffolds based on chromosome-level assemblies of the zebra finch genome (bTaeGut1.4.pri, GCF_003957565.2) and the Gambel’s white-crowned sparrow (*Z. l. gambelii*, GCF_028769735.1)^2^. This was applied to the macro-chromosomes, intermediate chromosomes and some micro-chromosomes. Thirty chromosomes were successfully assigned, including chromosomes 1, 1 A, 2–4, 4 A, 5–15, 17–28 and Z in NWCS (Fig. 1C). For the RUFS assembly, 27 chromosomes were assigned, comprising chromosomes 1, 1 A, 2–4, 4 A, 5–15, 17–22, 24, 26, 27 and Z (Fig. 1D). For any future modification, the details of chromosome assignment are provided in Table 3, 4. The karyotype of assigned chromosomes is shown in Supplementary file 1 (Figure S1). Other chromosomes, including micro-chromosomes and the W chromosome, remain unassigned or fragmented in the assemblies. Assembling micro-chromosomes in avian species has proven particularly challenging^27^ due to their small size, high GC content, and complex repetitive sequences. While we lack full confidence in the complete assignment of these chromosomes, we were able to illustrate scaffolds that partially represent these chromosomes, such as micro-chromosomes 31, 32, 35 and W chromosome (Figure S2).

**Table 3:** Chromosome assignment for the Nuttall’s white-crowned sparrow (*Z.l. nuttalli*) assembly.

Scaffold	Chromosome
NWCS_2	1
NWCS_5	1 A
NWCS_1	2
NWCS_3	3
NWCS_6	4
NWCS_15	4 A
NWCS_7	5
NWCS_9	6
NWCS_8	7
NWCS_10	8
NWCS_11	9
NWCS_14	10
NWCS_12	11
NWCS_13	12
NWCS_16	13
NWCS_17	14
NWCS_19	15
NWCS_21	17
NWCS_20	18
NWCS_22	19
NWCS_18	20
NWCS_23	21
NWCS_29	22
NWCS_25	23
NWCS_24	24
NWCS_30	25
NWCS_26	26
NWCS_27	27
NWCS_28	28
NWCS_4	Z

**Table 4:** Chromosome assignment for the rufous-collared sparrow (*Z. c. chilensis*) assembly.

Scaffold	Chromosome
ZC_2	1
ZC_5	1 A
ZC_1	2
ZC_3	3
ZC_6	4
ZC_15	4 A
ZC_7	5
ZC_9	6
ZC_8	7
ZC_10	8
ZC_11	9
ZC_14	10
ZC_19	11
ZC_13	12
ZC_16	13
ZC_17	14
ZC_20	15
ZC_23	17
ZC_22	18
ZC_24	19
ZC_18	20
ZC_25	21
ZC_29	22
ZC_26	24
ZC_27	26
ZC_28	27
ZC_4	Z

**Fig. 2:**
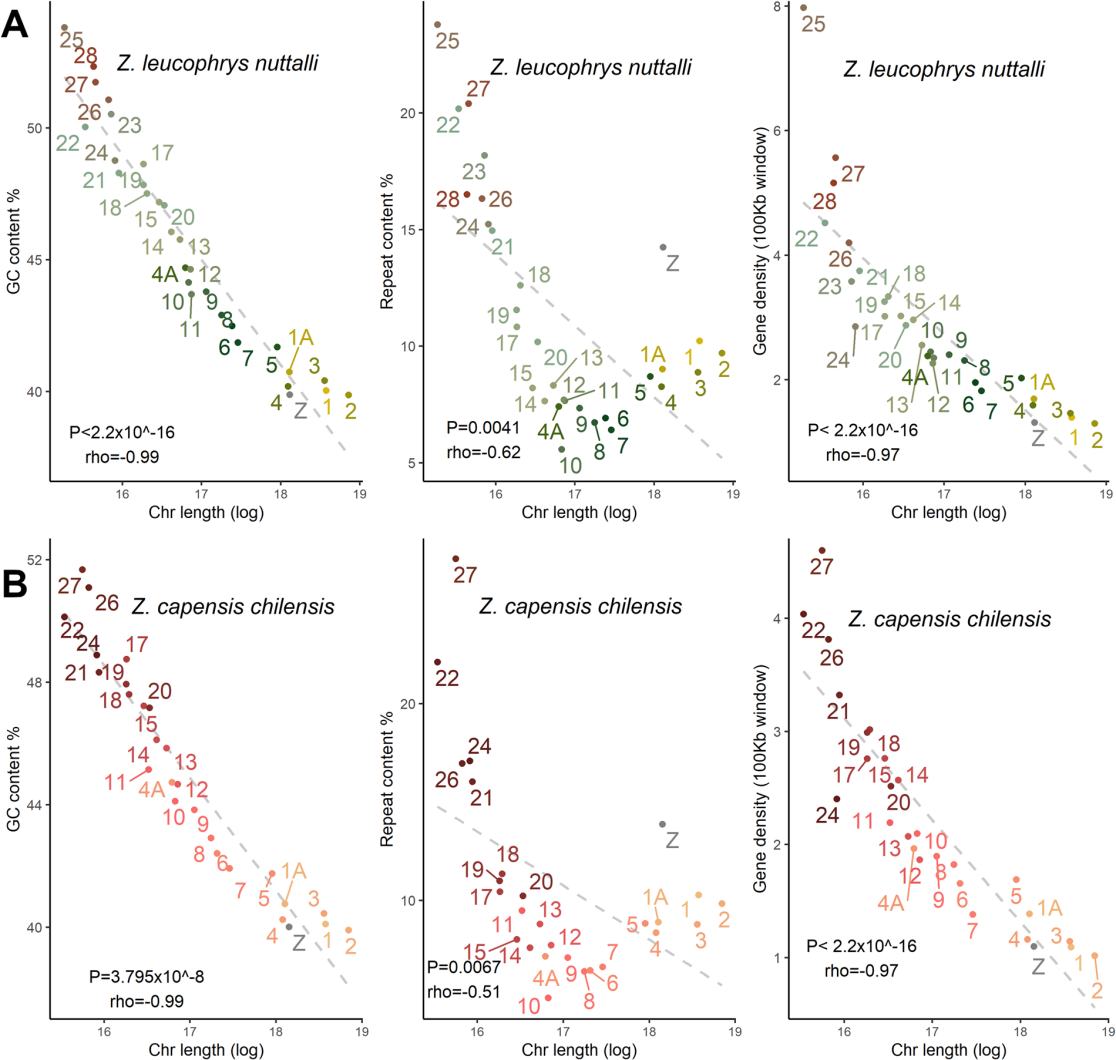
Correlation between chromosome size and sequence features of assemblies. Chromosome length (log transformed) is shown on the x-axis and GC content (%), repeat elements (%), and gene density (100 Kb window) are shown on the y-axis for (**A**) the white-crowned sparrow (*Zonotrichia leucophrys nuttalli*) genome and (**B**) the rufous-collared sparrow (*Zonotrichia capensis chilensis)* genome. The P value was calculated by Spearman’s test and the correlation coefficient is denoted by rho.

To further investigate the relationship between assembled chromosomes and sequence content, the length of chromosome is shown to be negatively correlated with its GC content, repeat content, and gene density (Fig. 2). To be specific, the smaller chromosomes, particularly the micro-chromosomes (e.g., chromosome 25 and 27), present greater complexity, which further adds to the challenge of assembling them accurately. This has been previously observed in other avian genomes, such as chicken and barn swallow (*Hirundo rustica*)^28^, as well as the Gambel’s white-crowned sparrow (*Z. l. gambelii*)^2^.

### RNA-seq sample preparation and sequencing

In order to predict gene structures, RNA samples were further sequenced to assist the gene model annotation. RNA sequencing was performed on the ovary, heart, liver and brain collected from an adult female NWCS (different individual to the one used for genome assembly). The NWCS was collected from a wild free-living population using mist nets under regular weather conditions at Point Reyes field site in California, USA. Samples were immediately flash frozen on dry ice and maintained frozen until being transferred to a −80 °C freezer in the laboratory at University of California Davis, USA. Approximately 100 mg to 150 mg of tissue was homogenized for RNA extraction. For the RUFS, the RNA-seq dataset available under accession number PRJNA298255^29^ was used, which includes a pectoral muscle sample sequenced on the Illumina HiSeq 2500 platform.

To extract mRNA for RNA-sequencing, tissues were homogenized in TRIzol reagent (Invitrogen, Waltham, USA) and the Direct-zol RNA Miniprep kit (Zymo Research, Irvine, USA) was used for RNA isolation. The RNA was eluted in RNAse-free water ensuring a concentration greater than 12.5 ng/µL, yielding a total of 500 ng of RNA. The library preparation was constructed with polyadenylated RNA (polyA) selection and sequenced on the BGI DNBSEQ platform, generating 150 bp paired-end reads with around 30 million sequences per sample. The soft-masked genome was first indexed by running the ‘--runMode genomeGenerate’ module in STAR (version 2.7.8a)^30^ with default options. Reads were then mapped to the genome and sorted with the ‘--outSAMtype BAM SortedByCoordinate’ option. The RNA-seq data were used as the transcriptomic evidence for the gene model annotation.

### Gene model annotation

Following the genome assembly, a gene model annotation was generated for the two *Zonotrichia* sparrow assemblies. Various sources of evidence were used within the BRAKER (v2.1.6) annotation pipeline^31^. In short, the ETP mode in BRAKER was implemented with RNA-seq evidence and protein homology information retrieved from closely-related reference species. RNA-seq evidence, consisted of data generated from four tissues for the NWCS (ovary, heart, liver and brain), and a publicly-available RNA-seq dataset from the pectoral muscle for the RUFS annotation. The protein hints included several sources, including OrthoDB vertebrate, chicken (GRCg6a) and zebra finch (bTaeGut1.4.pri) protein databases. The mapped RNA-seq evidence, together with the protein hints, were used for training GeneMark-ETP (version 4.71_lic)^32^. Subsequently, AUGUSTUS (version 3.4.0)^31,33^ was trained by using the same evidence on highly supported genes that had strong support and the existing ‘chicken’ config and parameters were employed for training purposes.

**Fig. 3:**
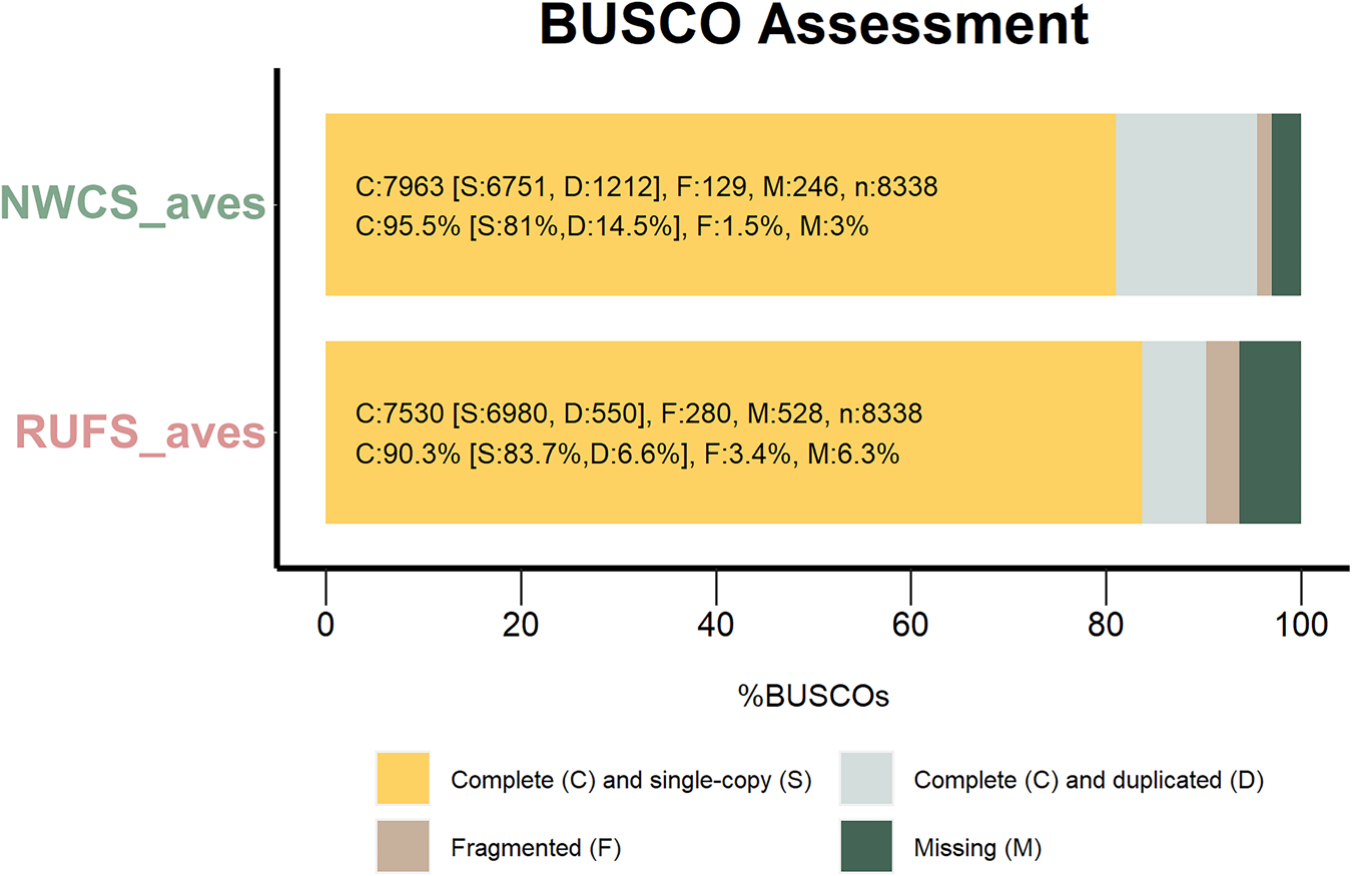
Benchmarking Universal Single-Copy Orthologs (BUSCO) assessments of the NWCS and RUFS transcriptome. The BUSCO analyses were performed using the ‘aves_odb10’ lineage dataset.

**Fig. 4:**
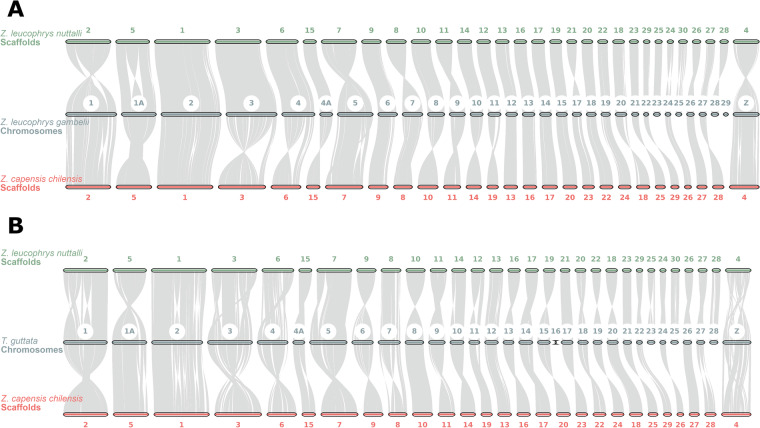
Synteny between genome assemblies. (**A**)The Macrosynteny plot shows alignments between the Nuttall’s white-crowned sparrow (*Z. leucophrys nuttalli*) and Gambel’s white-crowned sparrow (*Z. leucophrys gambelii*), as well as that between the rufous-collared sparrow (*Z. capensis chilensis*) and the Gambel’s white-crowned sparrow. The chromosome labels in the centre represent the Gambel’s white-crowned sparrow chromosomes (GCF_028769735.1), while the numbered labels indicate the scaffold numbering for *Z. leucophrys nuttalli* (top) and *Z. capensis chilensis* (bottom) assemblies. (**B**) Synteny between *Zonotrichia* genome assemblies and the zebra finch (*T. guttata*) genome.

In summary, the gene models annotated 21,727 and 17,845 genes for the NWCS and RUFS assemblies respectively, including 263,795 and 194,070 exons in total. The NWCS and RUFS annotations have an average gene length of 27,878 bp and 22,735 bp. The average exon lengths are 209 bp for NWCS and 253 bp for RUFS, with average intron lengths of 26,302 bp and 21,077 bp respectively (Table 5). In addition, Figure S3 shows the distribution of exon counts within genes for the two assemblies. Subsequently, the quality of annotations was assessed using several approaches. First, the completeness of annotation was assessed using BUSCO analysis (v5.2.2) for transcriptome using the ‘*aves*’ dataset. In brief, results showed that NWCS and RUFS transcriptomes consisted of 95.5% and 90.3% complete (C) BUSCOs, respectively (see more BUSCOs in Fig. 3). The RUFS annotation exhibited a less complete gene model compared to NWCS, likely due to the limited availability of RNA-seq evidence for annotation training. Future efforts incorporating RNA-seq data from additional tissues (e.g., brain and gonadal tissues) would significantly improve the annotation completeness. This confirmed that the two annotations were comprehensive and represented good quality. Furthermore, the protein coding potential of predicted transcripts was estimated using CPC2 (0.1)^34^, which can quickly generate accurate evaluation of the coding ability of transcripts in a species-neutral manner, especially for long non-coding transcripts. The coding probability of a transcript is estimated and classified into non-coding (0 - 0.5) or coding (0.5 - 1). In total, 3,709 non-coding transcripts were detected in the NWCS annotation, with 1,124 non-coding transcripts in the RUFS annotation. Next, the transfer RNA (tRNA) profile of the genomes was further predicted using tRNAscan-SE (v.2.0.12)^35^, enabling functional classification of small non-coding RNAs. In total, we identified 496 tRNAs for NWCS, and 469 for RUFS, which is very similar to that found in the closely-related GWCS genome assembly (495 tRNAs) (NCBI RefSeq: GCF_028769735.1)^2^. In addition, macro-synteny was established for the GWCS, NWCS and RUFS models, showing a total of 31 chromosomes in the GWCS assembly (Fig. 4A). For further details, refer to the *Technical Validation* section of this manuscript.

**Table 5:** Summary of gene annotation for the two *Zonotrichia* sparrow genomes.

Annotation summary	*Z. leucophrys nuttalli*	*Z. capensis chilensis*
Number of genes	21,727	17,845
Number of transcripts	25,755	19,396
Number of exons	263,795	194,070
Number of introns	237,972	174,659
Average length of genes(bp)	27,878	22,735
Average length of exons(bp)	209	253
Average length of introns(bp)	26,302	21,077
Predicted non-coding transcripts	3,709	1,124
tRNA	496	469

## Data Records

The data presented in this study is available in the National Center for Biotechnology Information (NCBI) repository. The two genome assemblies can be accessed under GCA_045281155.1 (*Z. l. nuttalli*)^36^ and GCA_045269165.1 (*Z. c. chilensis*)^37^. The RNA-seq datasets used for data validation are available under project accession numbers: SRP535723^38^ (*Z. l. nuttalli*) and SRP064664^29^ (*Z. c. chilensis*). The whole-genome sequencing data are found under project accession numbers: SRP536580^39^ (*Z. l. nuttalli*) and SRP536581^40^ (*Z. c. chilensis*).

## Technical Validation

The quality of the two genome assemblies was evaluated by aligning to the recently published reference genome assembly of the Gambel’s white-Crowned Sparrow (*Zonotrichia leucophrys gambelii*, GWCS); NCBI RefSeq: GCF_028769735.1^2^. To demonstrate the synteny of gene blocks between our genomes and the closely related GWCS species, the comparative genomic tool MCscan, implemented in the jcvi (1.4.16)^41^ libraries, was used to identify syntenic regions between the three genomes (GWCS, NWCS, and RUFS) using the ‘compara’ module. The module first performs a sensitive LAST^42^ pipeline to find orthologs and then filters for ‘C-score’ with ‘--cscore = 0.99’. Synteny blocks were then extracted with the ‘screen’ function with ‘--minspan=30’. Macrosynteny results were visualized with the ‘jcvi.graphics.karyotype’ module showing the chromosomes in the GWCS assembly. The depths in the synteny showed that around 90% of genes exhibit a one-to-one pattern in both assemblies (Figures S4, S5). Moreover, the NUCmer (NUCleotide MUMmer) aligner built in MUMmer (version 3.1)^43^ was used to perform whole genome alignments to complement the synteny analyses. The percentage of total bases aligned to GWCS was 97.14% and 96.16% for NWCS and RUFS, respectively. When compared to the GWCS annotation, the NWCS annotation has 20,938 commonly identified genes (96.37%), with 789 being uniquely found in NWCS. RUFS showed 17,459 (97.84%) commonly identified genes and 386 unique ones. These genes may suggest the fundamental and biological difference between the (sub-)species, however it should be noted that the completeness and quality of the gene annotation is largely influenced by many factors, such as the extrinsic evidence used for prediction, the fragmentation of contigs, and orientation of scaffolds. A synteny pattern between our genome assemblies and the zebra finch genome (bTaeGut1.4.pri) was also constructed (Fig. 4B).

In addition, the quality of our assemblies as reference genomes was evaluated by assessing their performance with whole-genome sequencing data and RNA-sequencing data. The whole-genome sequencing data were mapped to the final assembly using BWA-MEM (0.7.18)^44^ with default options. Mapping quality was evaluated using Qualimap (v2.3)^45^. The percentages of mapped reads were 98.66% for NWCS and 98.16% for RUFS, with average coverages of 44.69X and 57.39X, and average mapping qualities (MAPQ evaluates the probability of mapping mistakes) of 13.74 and 19.48, respectively. RNA-seq datasets were mapped to the genomes using STAR (V2.7.8a)^30^ with default parameters. Table 6 shows the summary of alignment quality, suggesting successful alignment with good quality. On average, the number of inputs was 34 million reads with a unique mapping rate of 92.62%. The average mapping length of read pairs was 295.69 bp with a mismatch rate of 0.80% per base.

**Table 6:** Summary of the Nuttall’s white-crowned sparrow RNA-seq mapping quality.

Sample type	Number of input reads	Uniquely mapped reads	Average mapping length (2 × 150 bp)	Mismatch rate per base
Gonad	33,951,110	92.11%	294.83	0.89%
Heart	33,657,723	90.77%	295.39	0.83%
Brain	34,349,521	93.43%	295.94	0.82%
Liver	34,018,583	94.15%	296.58	0.64%

### Ethics declarations

The work was approved by the Animal Welfare and Ethical Review Body at the Roslin Institute, The University of Edinburgh, UK and the University of California, Davis, USA Institutional Animal Care and Use Committee (AICUC) under protocol 19758, United States Fish and Wildlife Service - Federal MB90026B-0.

## Supplementary information

Supplementary file 1

## Data Availability

Analyses were conducted using standard bioinformatic tools (version and parameters as mentioned in Methods) running on the Scientific Linux system. This work has made use of the resources provided by the Edinburgh Compute and Data Facility (ECDF) (http://www.ecdf.ed.ac.uk/). Additional scripts used to process the results and generate the figures can be found in the GitHub repository: https://github.com/wzuhou/Zonotrichia_genomes_scripts/tree/main.
