# Supplementary material for: Genome assemblies of Nuttall’s White-crowned sparrow (Zonotrichia leucophrys nuttalli) and Rufous-collared sparrow (Zonotrichia capensis)
Source: Sci Data. 2025 Apr 3;12:567. doi: 10.1038/s41597-025-04889-x (PMC11968903; doi:10.1038/s41597-025-04889-x)
Supplement: Supplementary file 1 [file 41597_2025_4889_MOESM1_ESM.pdf]

Supplementary Figures

Corresponding author(s): Zhou Wu (zhou.wu@roslin.ed.ac.uk) and Jacqueline Smith (Jacqueline.smith@roslin.ed.ac.uk)

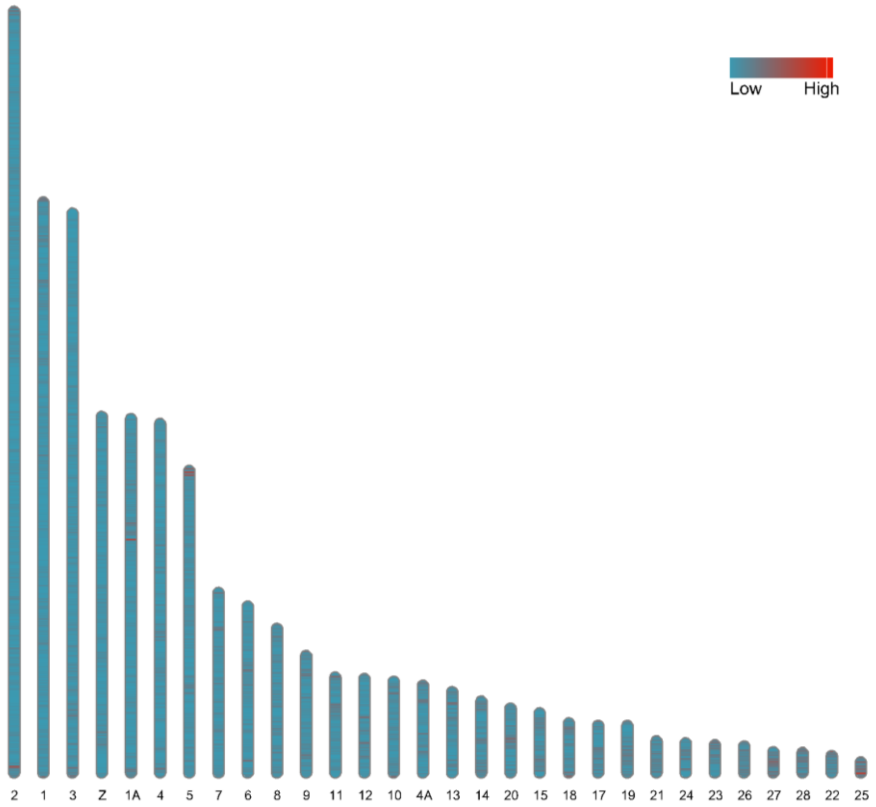

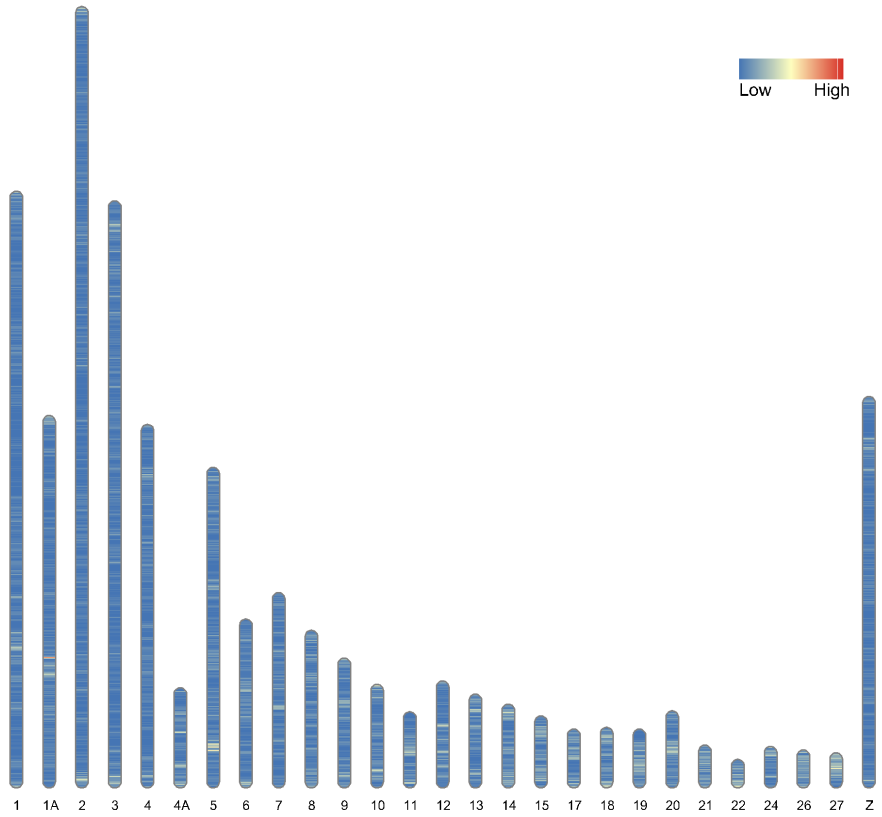

Figure S1. Chromosome assignments in the NWCS (top) and RUFS (bottom) genome assemblies, displayed with corresponding gene density. Only the scaffolds representing major chromosomes are shown, and unplaced scaffolds have been omitted.

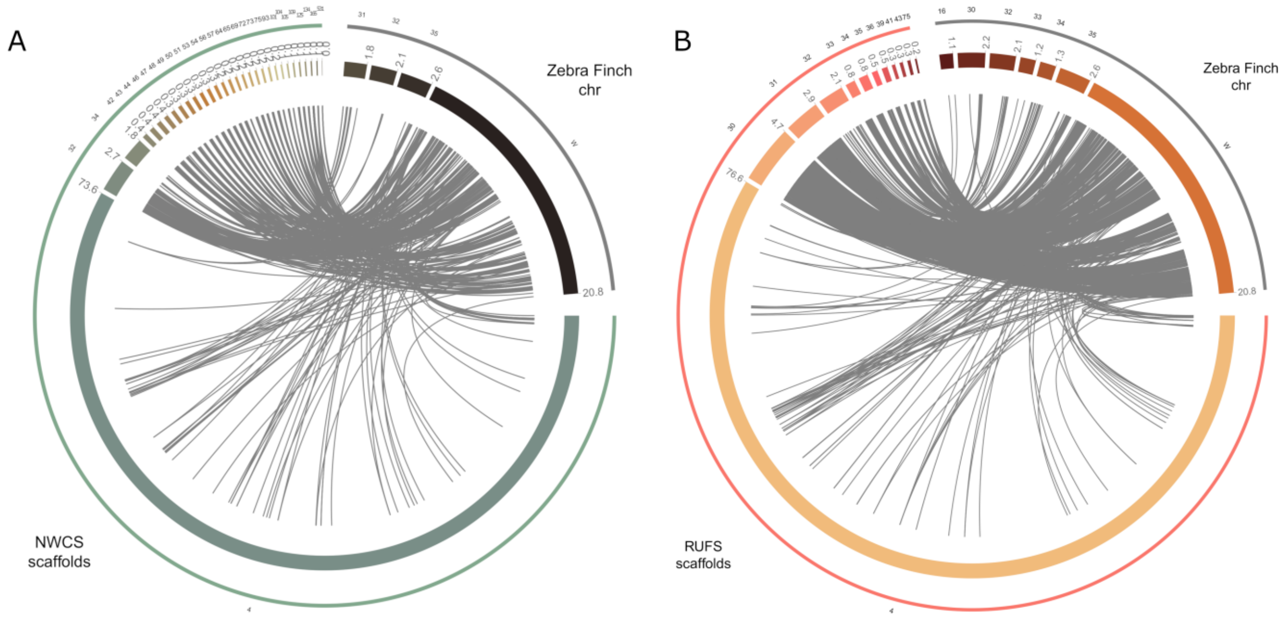

Figure S2. Alignment of the two *Zonotrichia* assemblies with zebra finch genome for the W and micro-chromosomes. (A) Alignment between the Nuttal's white-crowned sparrow (NWCS) and zebra finch, (B) as well as between the rufous-collared sparrow (RUFS) and zebra finch. Sequence lengths are indicated in Mb. Additionally, scaffold 4 in each assembly represents the Z chromosome, due to sequence homology, excessive alignment to the W chromosome was observed.

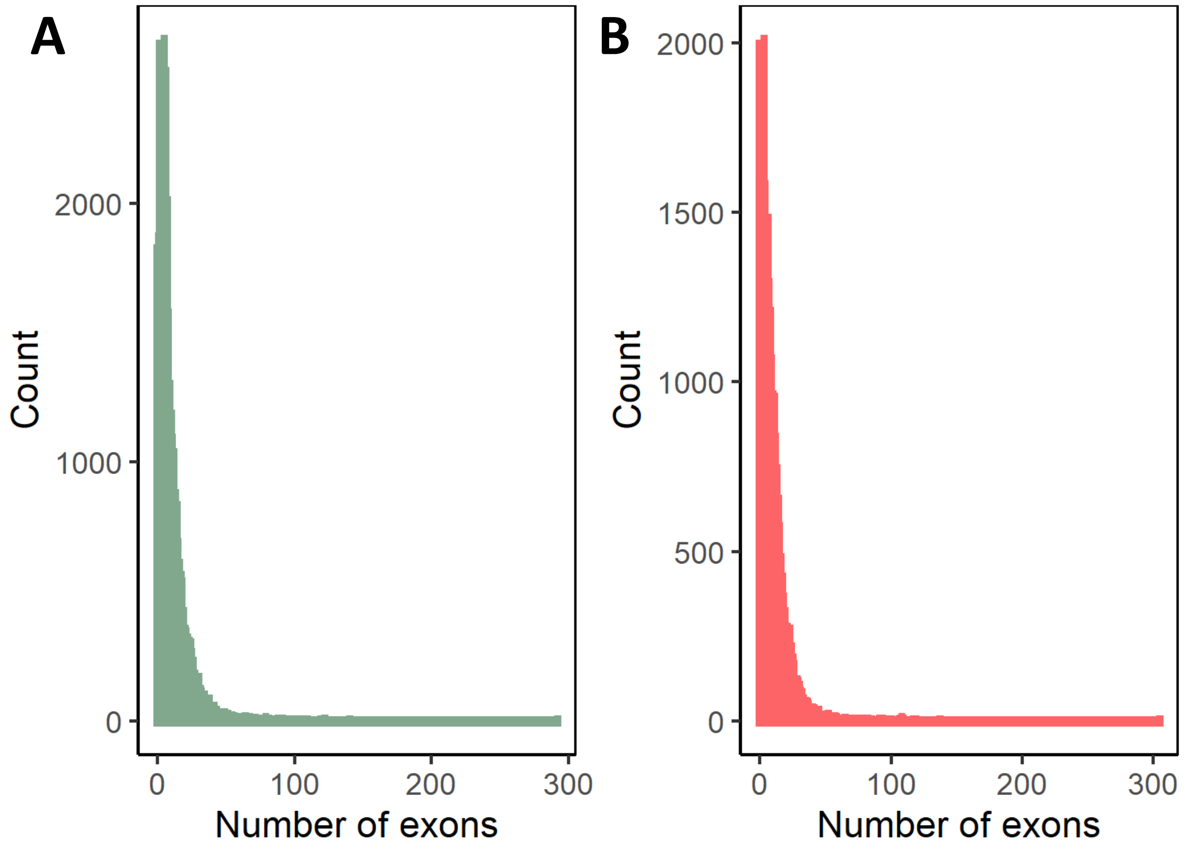

Figure S3. Histogram shows the number of exons within gene features for the *Zonotrichia* sparrow assemblies. The number of exons for (**A**) NWCS annotation, and (**B**) for RUFS annotation.

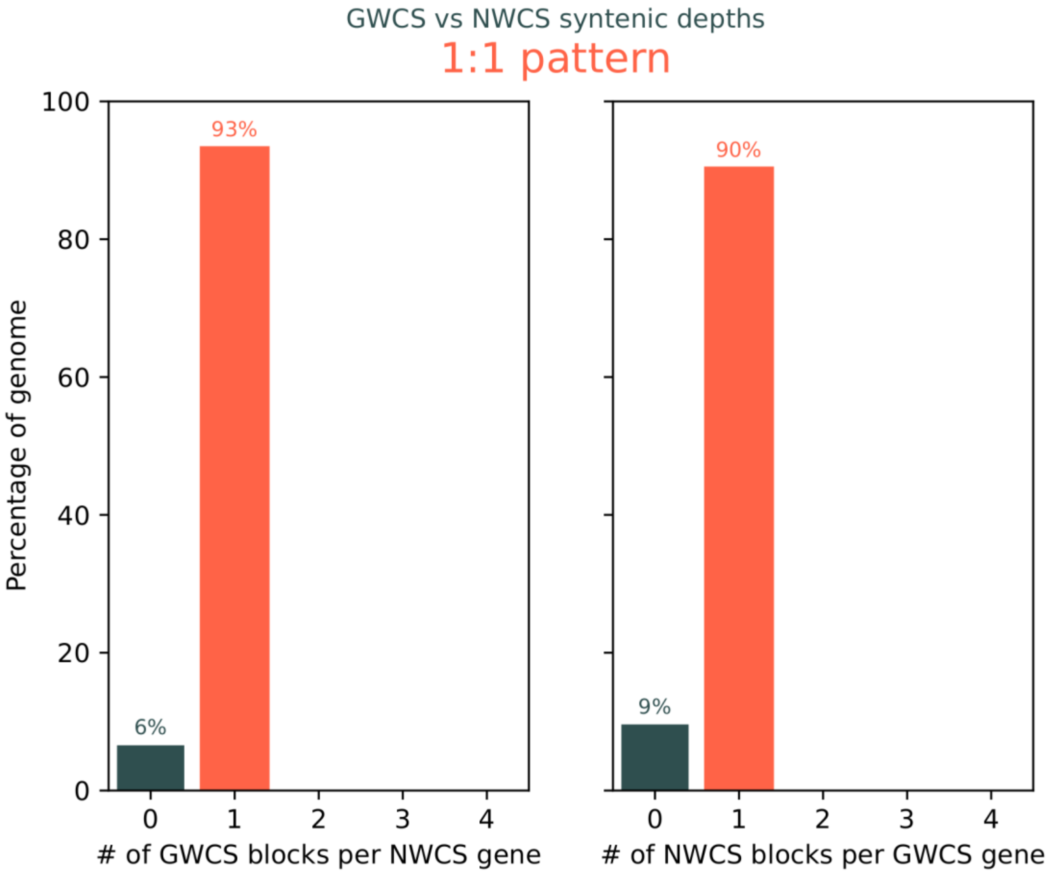

Figure S4. The depths of synteny between GWCS and NWCS.

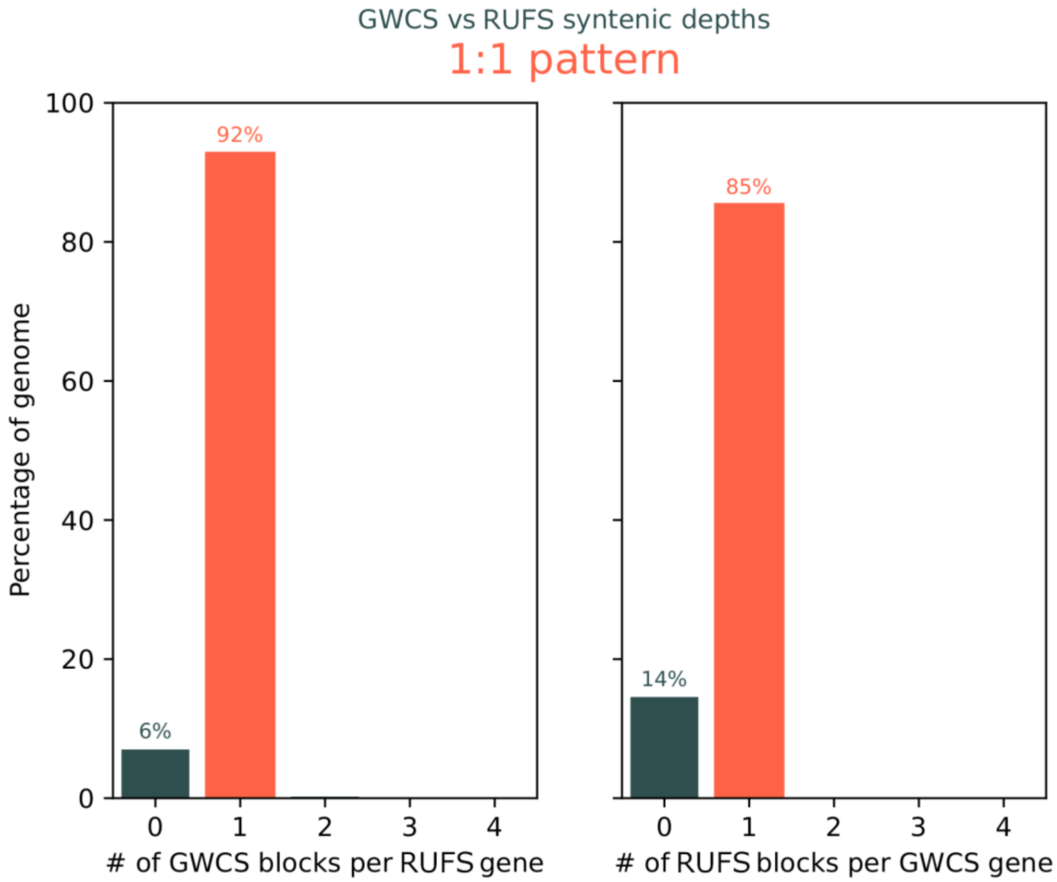

Figure S5. The depths of synteny between GWCS and RUFS.
